# A Mathematical Model for Biocontrol of the Invasive Weed *Fallopia japonica*

**DOI:** 10.1007/s11538-016-0195-8

**Published:** 2016-08-04

**Authors:** Stephen A. Gourley, Jing Li, Xingfu Zou

**Affiliations:** 1 Department of Mathematics, University of Surrey, Guildford, Surrey GU2 7XH UK; 2 Department of Mathematics, California State University Northridge, Northridge, CA 91330-8313 USA; 3 Department of Applied Mathematics, University of Western Ontario, London, ON N6A 5B7 Canada

**Keywords:** Delay differential equation, Age-structure, Bio-control, Invasive weed

## Abstract

We propose a mathematical model for biocontrol of the invasive weed *Fallopia japonica* using one of its co-evolved natural enemies, the Japanese sap-sucking psyllid *Aphalara itadori*. This insect sucks the sap from the stems of the plant thereby weakening it. Its diet is highly specific to *F. japonica*. We consider a single isolated knotweed stand, the plant’s size being described by time-dependent variables for total stem and rhizome biomass. It is the larvae of *A. itadori* that damage the plant most, so the insect population is described in terms of variables for the numbers of larvae and adults, using a stage-structured modelling approach. The dynamics of the model depends mainly on a parameter *h*, which measures how long it takes for an insect to handle (digest) one unit of *F. japonica* stem biomass. If *h* is too large, then the model does not have a positive equilibrium and the plant biomass and insect numbers both grow together without bound, though at a lower rate than if the insects were absent. If *h* is sufficiently small, then the model possesses a positive equilibrium which appears to be locally stable. The results based on our model imply that satisfactory long-term control of the knotweed *F. japonica* using the insect *A. itadori* is only possible if the insect is able to consume and digest knotweed biomass sufficiently quickly; if it cannot, then the insect can only slow down the growth which is still unbounded.

## Introduction

The Japanese knotweed *Fallopia japonica* has been present in the UK since 1825. It was originally introduced by the Victorians as an ornamental plant, but soon escaped from their gardens. The plant can grow extremely quickly, forming very dense thickets which can shade out native plants and offer a poor habitat for native insects, birds and mammals, can grow in any soil type, and can penetrate concrete and cause damage to paved areas. DEFRA estimates the total cost of control of the weed in the UK to be £1.56 billion (see DEFRA 2003). One of the main problems with the plant is that it has no natural predators in the UK and therefore does not compete fairly with native species. Swansea is one of the worst affected areas, and it has been estimated that the current infestation there would cost $\pounds$ 9.5 million to treat completely (see Cardiff 2006). The plant is also found in most of the states of the USA and several Canadian provinces.

The main quantifiable cost is herbicide treatment, but the weed has an extensive root system and can penetrate paving, tarmac and asphalt. It can damage flood defence structures and archaeological sites, and its removal can significantly add to the costs of preparing development sites. The weed is very difficult to eradicate and efforts at removal involving disturbing the soil can make matters worse. It forms very dense thickets that reduce native species diversity and are of little value to wildlife. It spreads easily: fragments of stem and rhizome are easily conveyed along rivers to distant sites, and the fibrous stems are slow to decompose. Reproduction of Japanese knotweed in the UK is currently by vegetative means only (regeneration from root material), and dispersal is due primarily to rhizome fragments being washed downstream in rivers, or the transportation by humans of soil containing rhizome fragments. Sexual reproduction does not occur in the UK as only the female plant is present there. In the native habitat, insect pollination, sexual reproduction, and wind dispersal of seed can also be contributing factors. Experimental work in the USA by Forman and Kesseli (2003) suggests considerable implications for management of the weed, should sexual reproduction start to become important.

Currently, control of *F. japonica* is primarily by chemical means using a herbicide such as glyphosate. This carries risks of contamination of rivers and streams where many knotweed areas are found, and in any case knotweed is in such rapid expansion that increased chemical use is not a long-term option. The shoots are edible by sheep, goats, cattle and horses and therefore satisfactory control is already achievable in grazing locations.

In this paper, we propose a mathematical model for the biocontrol of *F. japonica* using one of its co-evolved natural enemies, the Japanese sap-sucking psyllid *Aphalara itadori*. This insect sucks the sap from the stems of the plant thereby weakening it. After extensive experimental trials by the agricultural research organisation CABI (2007), in 2010 DEFRA in the UK approved the release of this insect as a biocontrol agent for *F. japonica*, initially at a handful of closely monitored sites. The insect was selected, among numerous species of plant-eating insects and fungi, because its diet is highly specific to *F. japonica*. Biological control has the advantage of being environmentally sound and more sustainable than extensive herbicide use, but the strategy will not result in complete eradication. The most that can be anticipated is that an equilibrium is reached in which the knotweed is still present but at an acceptable level. Such a strategy has been highly successful in controlling the Salvinia weed in Sri Lanka which was reduced by 80 % in four years by the introduction of the weevil *Cyrtobagous salviniae*, and in Kerala, India (Jayanth 1987). In the UK it seems likely that all knotweed is of one single clone (Hollingsworth and Bailey 2000), and the absence of sexual reproduction makes the plant a good candidate for biological control.

The model we develop in this paper is for a single isolated knotweed stand, and the plant’s size is described by time-dependent variables for total stem and rhizome biomass. Spatial effects are not modelled explicitly. However, the stems only grow to a certain height (about 3 m) and the roots only to a certain depth, also about 3 m. Therefore, unlimited growth of plant biomass, one of the predictions of our model under certain conditions, can only mean spatial spread of the stand. As far as the insects are concerned, it is the larvae of *A. itadori* that do the most damage to the plant and so the insect population is broken down into numbers of larvae and adults, using a standard stage-structured modelling approach. We assume that only larvae eat the stems (more precisely, the sap from the stems). Information on *A. itadori* can be found in Shaw et al. (2009). Eggs are laid on the upper surfaces of leaves, and the egg to adult duration is around 33 days (knotweed can grow one metre in this time). Insects can overwinter on sheltering plants. Neither larvae nor adults of *A. itadori* will eat the roots. Another species tested by CABI, the endoclyta moth, does eat the roots but was rejected as a biocontrol agent because it was found to also attack another unrelated plant (CABI 2007).

In this paper we want to give due consideration to the fact that the knotweed plant species exhibits unusual growth behaviour due to the fact that it is an invasive species that grows without the usual inhibiting factors of predation, or even lack of physical space. Given enough land, there is no limit to the extent to which it can spread over the time scale of interest and therefore even exponential growth is not unrealistic. The reader will notice that the way we model stem and rhizome growth does not provide for a carrying capacity and in fact the production rates for these quantities are linear. This is intentional and, far from being a simplification, it actually makes the model remarkably resistant to analysis that would usually lead in a straightforward way to properties such as boundedness of solutions, extinction or persistence using well-developed theories. The overall model is nonlinear, due to the fact that although the number of knotweed stems may grow without bound, each stem only grows to a finite height and therefore has a finite carrying capacity for eggs. This is modelled using a nonlinear function that represents the egg laying rate per stem, which levels off and may even decrease as the number of adults per stem increases due to crowding effects.

It turns out that the dynamics of the model depends mainly on a parameter *h* in our model, which measures how long it takes for an insect to handle (digest) one unit of *F. japonica* stem biomass. If *h* is too large then the model does not have a positive equilibrium and the plant biomass and insect numbers both grow together without bound, though at a lower rate than if the insects were absent. On the other hand, if *h* is sufficiently small, then the model possesses a positive equilibrium which appears to be locally stable. The structure of the model and its lack of monotonicity properties make it remarkably resistant to analytic study and therefore the analysis is based mostly on linear theory and backed up with numerical simulation. The overall take home message is simple: satisfactory long-term control of the knotweed *F. japonica* using the insect *A. itadori* is only possible if the insect is able to consume and digest knotweed biomass sufficiently quickly; if it cannot, then the insect can only slow down the growth which is still unbounded.

**Table 1:** Parameters used in the model and simulations

Symbol	Meaning	Value	Reference
$\tau _{\mathrm{p}}$	Development time of *A. itadori* from egg to adult (maturation time)	32.2 ± 0.5 days (28–42 days)	Shaw et al. (2009)
$\tau _\mathrm{s}$	Life-span of a stem	150 days	Approximation
$k_\mathrm{l}$	Additional per-capita larval *A. itadori* mortality when sap is in short supply	$k_\mathrm{l}=1-m$	
$m=\mu _\mathrm{l}^{\infty }$	Natural per-capita larval *A. itadori* mortality	0.0205 per day	Shaw et al. (2009)
	When sap is plentiful		
*P*	Maximum number of eggs per unit time that an individual adult can lay	23.9798 eggs per adult per day	Shaw et al. (2009)
*q*	Crowdedness constant	1	Approximation
$\mu _{\mathrm{a}}$	Per-capita mortality of adult *A. itadori*	0.0806 per day	Shaw et al. (2009)
$\mu _\mathrm{s}$	Per-capita natural mortality of stem biomass	0.01 per day per unit	Approximation
*e*	Resource (sap) encounter rate	1	Approximation
*h* ($h_{\mathrm{crit}}$)	Handling (digestion) time per unit biomass consumed	The number of days per unit per larva	(To be estimated)
$\sigma$	Fraction of encountered food biomass that a larvae ingests	1	Approximation
$k_\mathrm{s}$	Birth rate for stems	0.1 unit per day	Approximation
$k_\mathrm{r}$	Birth rate for rhizome	0.1 unit per day	Approximation
$\mu _\mathrm{r}$	Per-capita loss rate of rhizome biomass	0.01 per day per unit	Approximation
$A^0$	Initial number of adult *A. itadori*	50	Approximation
$R^0$	Initial quantity of rhizome biomass	500 units	Approximation

## Model Derivation

For a complete list of notation, and the values given to parameters later in this paper, see Table 1. The state variables are *A*(*t*), *L*(*t*), *S*(*t*) and *R*(*t*), where *A*(*t*) and *L*(*t*) are the numbers of adult and larval *A. itadori* on a particular knotweed stand at time *t*, *S*(*t*) is the total stem biomass of the knotweed stand, and *R*(*t*) is total rhizome biomass. We begin by deriving equations that govern the evolution of these variables, starting with the larval *A. itadori*. Let *a* denote age and *l*(*t*, *a*) denote the age density of the larvae. Let $\tau _\mathrm{p}$ (the subscript p standing for psyllid) denote the developmental time of *A. itadori* from egg to adult. Using a standard age-structured modelling approach, we may write

1 $$\begin{aligned} \frac{\partial l(t,a)}{\partial t}+\frac{\partial l(t,a)}{\partial a} = -\mu _\mathrm{l}(S(t))l(t,a), 0<a<\tau _\mathrm{p} \end{aligned}$$

which simply expresses the fact that larval *A. itadori* are lost at a per-capita rate $\mu _\mathrm{l}(S(t))$ that depends on the total stem biomass. The function $\mu _\mathrm{l}(\cdot )$ will be decreasing, since larval mortality will be greater if there is a shortage of stems, and hence of sap, and is assumed to approach a positive limit $\mu _\mathrm{l}^{\infty }$ as its argument tends to infinity, that limit being the natural per-capita mortality when there is no shortage of sap. We also write $\mu _\mathrm{l}^0=\mu _\mathrm{l}(0)$.

For $t>a$ define $l_{\xi }(a)=l(a+\xi ,a)$. Differentiation shows that

$$\begin{aligned} \frac{\hbox {d}l_{\xi }(a)}{\hbox {d}a}=-\mu _\mathrm{l}(S(a+\xi ))l_{\xi }(a). \end{aligned}$$

Therefore

$$\begin{aligned} l_{\xi }(a)=l_{\xi }(0)\exp \left( -\int _0^a\mu _\mathrm{l}(S(\eta +\xi ))\,\hbox {d}\eta \right) \end{aligned}$$

and, setting $$\xi =t-a$$,

2 $$\begin{aligned} l(t,a)=l(t-a,0)\exp \left( -\int _{t-a}^t\mu _\mathrm{l}(S(\eta ))\,\hbox {d}\eta \right) . \end{aligned}$$

Now, *l*(*t*, 0) is the birth rate of *A. itadori*, which should depend on the population of adult *A. itadori* and on the biomass of the knotweed stand available. Note that an individual stem can only accommodate a certain number of eggs so that intra-specific competitive effects apply at the level of the individual stem. Due recognition of this fact is crucial, since an important aspect we wish to study is the possibility that the knotweed stand *as a whole* can grow without bound, and therefore so can the number of larval and adult *A. itadori*. To reflect such an important competitive effect, we let $b_\mathrm{p}(A(t)/S(t))$ be the egg laying rate *per stem* which is a function of the number of adult *A. itadori* per stem. We will keep $b_\mathrm{p}(\cdot )$ as general as possible, but it is useful to keep in mind the frequently used prototype $b_\mathrm{p}(x)=Px e^{-qx}$ where $P,q>0$ (the well-known Nicholson’s blowflies birth rate), and where *P* is the maximum number of eggs per unit time that an individual adult can lay. This choice models the idea that the egg laying rate per stem increases nearly linearly with the number of adults per stem if the latter is fairly low, but reaches a maximum and then rapidly drops off if the number of adults per stem is very high, since then the issue of available space on the leaves for oviposition becomes important. The birth rate of *A. itadori* is therefore given by

3 $$\begin{aligned} l(t,0)=S(t)b_\mathrm{p}\left( \frac{A(t)}{S(t)}\right) . \end{aligned}$$

If $b_\mathrm{p}(\cdot )$ is chosen as $b_\mathrm{p}(x)=Px e^{-qx}$, the implication is that the overall egg laying rate (i.e. for the whole knotweed stand) is $l(t,0)=PA(t)e^{-qA(t)/S(t)}$. This gives the expected formula *PA*(*t*) if there are very few adults per stem, and a much lower value if *A*(*t*) / *S*(*t*) is very large. Note also that $l(t,0)=0$ if $S(t)=0$ which again is correct because if there are no stems there are no leaves and no oviposition sites, and the factor $e^{-qA(t)/S(t)}$ measures the impact of crowdedness on egg laying.

Using (3), (2) becomes

4 $$\begin{aligned} l(t,a)=S(t-a)b_\mathrm{p}\left( \frac{A(t-a)}{S(t-a)}\right) \exp \left( -\int _{t-a}^t\mu _\mathrm{l}(S(\eta ))\,\hbox {d}\eta \right) \end{aligned}$$

and the total number of larvae is

5 $$\begin{aligned} L(t)= & {} \int _0^{\tau _\mathrm{p}}l(t,a)\,\hbox {d}a =\int _0^{\tau _\mathrm{p}}S(t-a)b_\mathrm{p}\left( \frac{A(t-a)}{S(t-a)}\right) \exp \left( -\int _{t-a}^t\mu _\mathrm{l}(S(\eta ))\,\hbox {d}\eta \right) \hbox {d}a \nonumber \\= & {} \int _{t-\tau _\mathrm{p}}^{t} S(\xi )b_\mathrm{p}\left( \frac{A(\xi )}{S(\xi )}\right) \exp \left( -\int _{\xi }^t\mu _\mathrm{l}(S(\eta ))\,\hbox {d}\eta \right) \hbox {d}\xi . \end{aligned}$$

Differentiating this yields

6 $$\begin{aligned} \begin{array}{rcl} \displaystyle {\frac{\hbox {d}L(t)}{\hbox {d}t}} &{} = &{} -\mu _\mathrm{l}(S(t))L(t)+S(t)b_\mathrm{p}\left( \displaystyle {\frac{A(t)}{S(t)}}\right) \\ &{}&{}-S(t-\tau _\mathrm{p})b_\mathrm{p}\left( \displaystyle {\frac{A(t-\tau _\mathrm{p})}{S(t-\tau _\mathrm{p})}}\right) \exp \left( -\displaystyle {\int _{t-\tau _\mathrm{p}}^t}\mu _\mathrm{l}(S(\eta ))\,\hbox {d}\eta \right) . \end{array} \end{aligned}$$

The last term in (6) is the rate at which larvae mature into adults. We may therefore immediately write down a differential equation for the number *A*(*t*) of adult *A. itadori*:

7 $$\begin{aligned} \frac{\hbox {d}A(t)}{\hbox {d}t}=S(t-\tau _\mathrm{p})b_\mathrm{p}\left( \frac{A(t-\tau _\mathrm{p})}{S(t-\tau _\mathrm{p})}\right) \exp \left( -\displaystyle {\int _{t-\tau _\mathrm{p}}^t}\mu _\mathrm{l}(S(\eta ))\,\hbox {d}\eta \right) -\mu _{\mathrm{a}} A(t) \end{aligned}$$

where $\mu _{\mathrm{a}}$ is the per-capita mortality of adult *A. itadori*.

We now derive an equation for the biomass *S*(*t*) of a stand. Shoots appear from the ground and rapidly form canes which are fully grown by the Summer and die back after that. The stems have their sap consumed by the larvae of *A. itadori*, and we assume this is the most important factor involved in stem damage, but we also allow for natural mortality of stem biomass, at a per-capita rate $\mu _\mathrm{s}$. Letting *s*(*t*, *a*) be the age density of stem biomass, we may suppose that stem loss is governed by

8 $$\begin{aligned} \frac{\partial s(t,a)}{\partial t}+\frac{\partial s(t,a)}{\partial a} = -\frac{e\sigma s(t,a)L(t)}{1+he\sigma S(t)} -\mu _\mathrm{s} s(t,a) \end{aligned}$$

where the first term in the right-hand side represents loss of stem biomass due to predation by *A. itadori* larvae. This is modelled essentially in terms of a Holling type II functional response. If stem biomass is very high, the sap consumption rate per larva would have to reach a plateau [the reader may wish to look ahead to see how the functional response eventually appears in the equation for *S*(*t*); see (12)]. In (8), the Holling type II functional response has been implemented with commonly used notation where *e* is the resource (sap) encounter rate, *h* is the handling (digestion) time per unit biomass consumed and $\sigma$ is the fraction of encountered food biomass that the larva ingests. Using similar calculations to those that led to the expression for *l*(*t*, *a*) in (4), we can show that

9 $$\begin{aligned} s(t,a)=s(t-a,0)e^{-\mu _\mathrm{s} a}\exp \left( -\int _{t-a}^t\frac{e\sigma L(\eta )}{1+he\sigma S(\eta )}\,\hbox {d}\eta \right) . \end{aligned}$$

Now, *s*(*t*, 0) is the rate of production of stem biomass, i.e. of new shoots. These originate from the roots and so we let $b_\mathrm{s}(R(t))$ be the production rate of stem biomass, where the subscript *s* stands for stems, and write

10 $$\begin{aligned} s(t,0)=b_\mathrm{s}(R(t)). \end{aligned}$$

Using (10) in (9), we find that the total stem biomass $S(t)=\int _0^{\tau _\mathrm{s}}s(t,a)\,\hbox {d}a$ is, after a change in the variable of integration, given by

11 $$\begin{aligned} S(t)=\int _{t-\tau _\mathrm{s}}^t b_\mathrm{s}(R(\xi ))e^{-\mu _\mathrm{s}(t-\xi )} \exp \left( -\int _{\xi }^t\frac{e\sigma L(\eta )}{1+he\sigma S(\eta )}\,\hbox {d}\eta \right) \hbox {d}\xi \end{aligned}$$

where $\tau _\mathrm{s}$ is the life-span of a stem, i.e. the time between the appearance of the new shoot and the time when the stem dies back. Differentiating (11) shows that

12 $$\begin{aligned} \begin{array}{rcl} S'(t) &{} = &{} -\left( \mu _\mathrm{s}+\displaystyle {\frac{e\sigma L(t)}{1+he\sigma S(t)}}\right) S(t)+b_\mathrm{s}(R(t)) \\ &{}&{} -e^{-\mu _\mathrm{s}\tau _\mathrm{s}}\exp \left( -\int _{t-\tau _\mathrm{s}}^t\frac{e\sigma L(\eta )\,\hbox {d}\eta }{1+he\sigma S(\eta )} \right) b_\mathrm{s}(R(t-\tau _\mathrm{s})). \end{array} \end{aligned}$$

The first term in the right hand side of (12) represent the loss of stems, either through natural loss or as a consequence of their sap having been sucked by larval *A. itadori*. The $b_\mathrm{s}(R(t))$ term is the rate of production of new shoots, and the last term represents the death, at time *t*, of stems which were new shoots at time $t-\tau _\mathrm{s}$ and survived both natural loss and *A. itadori* activity over the time period $[t-\tau _\mathrm{s},t]$. The exponential coefficients in that term represent the probabilities of surviving each of these forms of death over that time period.

Finally, we need an equation for the variable *R*(*t*) representing the knotweed rhizome biomass. One of the disadvantages of biocontrol using *A. itadori* is that the predator does not attack the root system which is strong and readily grows into new plants. Therefore, complete eradication cannot be expected. The root system is extensive can grow horizontally reaching up to 7 m from the parent plant, and be up to 3 m deep. Carbohydrates, made in the leaves, are carried back to the rhizome system which can store large quantities and are used for both top (stem) growth and root growth. We allow for loss of rhizome biomass at a per-capita rate $\mu _\mathrm{r}$, the subscript standing for rhizome or root. Given the resilience of the rhizome system of *F. japonica*, the value of $\mu _\mathrm{r}$ is likely to be small. We let $b_\mathrm{r}(S(t))$ be the production rate of new rhizome biomass, which is taken to be a function of stem biomass *S*(*t*) since root growth depends on the production of carbohydrate by photosynthesis in the leaves, and leaf biomass is directly related to stem biomass. We propose the following simple equation for rhizome biomass *R*(*t*):

13 $$\begin{aligned} R'(t)=b_\mathrm{r}(S(t))-\mu _\mathrm{r} R(t). \end{aligned}$$

## Model Analysis

The model to be solved consists of Eqs. (6), (7), (12) and (13) subject to the initial conditions

14 $$\begin{aligned} L(\theta )= & {} L^0(\theta )\ge 0,\quad \theta \in [-\tau _\mathrm{s},0], \nonumber \\ A(\theta )= & {} A^0(\theta )\ge 0,\quad \theta \in [-\tau _\mathrm{p},0], \nonumber \\ S(\theta )= & {} S^0(\theta )\ge 0,\quad \theta \in [-\max (\tau _\mathrm{p},\tau _\mathrm{s}),0], \nonumber \\ R(\theta )= & {} R^0(\theta )\ge 0,\quad \theta \in [-\tau _\mathrm{s},0], \nonumber \\ L^0(0)= & {} \int _{-\tau _\mathrm{p}}^{0} S^0(\xi )b_\mathrm{p}(A^0(\xi )/S^0(\xi )) \exp \left( -\int _{\xi }^0\mu _\mathrm{l}(S^0(\eta ))\,\hbox {d}\eta \right) \hbox {d}\xi , \nonumber \\ S^0(0)= & {} \int _{-\tau _\mathrm{s}}^0 b_\mathrm{s}(R^0(\xi ))e^{\mu _\mathrm{s}\xi } \exp \left( -\int _{\xi }^0\frac{e\sigma L^0(\eta )}{1+he\sigma S^0(\eta )}\,\hbox {d}\eta \right) \hbox {d}\xi . \end{aligned}$$

The last two equations in (14) are constraints on initial data which reflect a compatibility with (5) and (11). Non-negativity for all time holds only for initial data satisfying the two constraints, but the use of non-negative initial data that does not satisfy the two constraints would have only a transient effect on the dynamics.

In view of the apparent capability of a knotweed stand to grow in an unlimited manner in the absence of any form of control, it seems not at all unreasonable to consider the situation when the birth rate functions for stems and rhizome are taken as linear, and so we take

15 $$\begin{aligned} b_\mathrm{s}(R)=k_\mathrm{s} R,b_\mathrm{r}(S)=k_\mathrm{r} S. \end{aligned}$$

### Unbounded Knotweed Growth in Absence of *A. itadori*

If *A. itadori* is absent, then $A(t)=L(t)=0$, the equations for the knotweed stem and rhizome biomass *S*(*t*) and *R*(*t*) reduce to a simpler form from which we can obtain the following single equation for *R*(*t*):

16 $$\begin{aligned} R'(t)=-\mu _\mathrm{r} R(t)+b_\mathrm{r}\left( \int _{t-\tau _\mathrm{s}}^t b_\mathrm{s}(R(\xi ))e^{-\mu _\mathrm{s}(t-\xi )}\hbox {d}\xi \right) \end{aligned}$$

where we have used the integral expression (11) for *S*(*t*), with $L(t)=0$. With the choices (15), Eq. (16) becomes, after a substitution,

17 $$\begin{aligned} R'(t)=-\mu _\mathrm{r} R(t)+k_\mathrm{r}k_\mathrm{s}\int _{0}^{\tau _\mathrm{s}}R(t-\eta )e^{-\mu _\mathrm{s}\eta }\hbox {d}\eta . \end{aligned}$$

Solutions of the form $R(t)=\exp (\lambda t)$ exist whenever $\lambda$ satisfies a characteristic equation. Since the delay term in (17) has a positive coefficient and only involves finite delay, we may apply the theory in Smith (1995) to conclude that the dominant eigenvalue is real. The characteristic equation is

18 $$\begin{aligned} \lambda +\mu _\mathrm{r}=k_\mathrm{r}k_\mathrm{s}\left( \frac{1-e^{-(\lambda +\mu _\mathrm{s})\tau _\mathrm{s}}}{\lambda +\mu _\mathrm{s}}\right) \end{aligned}$$

and we may consider only its real roots to determine whether *R*(*t*) grows or decays with time. The ratio in round brackets is decreasing in $\lambda$ and simple calculus arguments show that the dominant real root of (18) is positive, so that the rhizome biomass *R*(*t*) grows exponentially, if $\mu _\mathrm{r}\mu _\mathrm{s}<k_\mathrm{r}k_\mathrm{s}(1-e^{-\mu _\mathrm{s}\tau _\mathrm{s}})$. If the reversed inequality holds, then it decays exponentially. We have proved the following result.

#### Theorem 3.1

Suppose that there are no A. itadori and that $b_\mathrm{s}(R)$ and $b_\mathrm{r}(S)$ are chosen as in (15). Then, if $\mu _\mathrm{r}\mu _\mathrm{s}<k_\mathrm{r}k_\mathrm{s}(1-e^{-\mu _\mathrm{s}\tau _\mathrm{s}})$ the knotweed stand grows exponentially, while if $\mu _\mathrm{r}\mu _\mathrm{s}>k_\mathrm{r}k_\mathrm{s}(1-e^{-\mu _\mathrm{s}\tau _\mathrm{s}})$ then it decays exponentially.

From this very simple analysis, we may make some useful observations. The stand grows if $k_\mathrm{r}k_\mathrm{s}$ is sufficiently large, as expected, and also if $\mu _\mathrm{r}$ is sufficiently small (which is likely to be the case, since roots are hard to destroy). But the dependence on $\mu _\mathrm{s}$ is more complicated. Note that, if $\mu _\mathrm{s}$ is very small, then the condition for exponential growth is approximated by $\mu _\mathrm{r}<\tau _\mathrm{s}k_\mathrm{r}k_\mathrm{s}$ which only holds if $\tau _\mathrm{s}$ is sufficiently large, i.e. stems live long enough before they die back. So, smallness of $\mu _\mathrm{s}$ is not by itself sufficient for the plant’s survival (but smallness of $\mu _\mathrm{r}$ is). It is hardly surprising that the plant has evolved to have such tough roots that are difficult to destroy.

### Impossibility of Eradication When $\mu _\mathrm{r}\mu _\mathrm{s}<k_\mathrm{r}k_\mathrm{s}(1-e^{-\mu _\mathrm{s}\tau _\mathrm{s}})$

We prove the following result which establishes that, if the knotweed stand grows exponentially in the absence of *A. itadori*, then it cannot be eradicated in the presence of *A. itadori*.

#### Theorem 3.2

Suppose that $b_\mathrm{s}(R)$ and $b_\mathrm{r}(S)$ are chosen as in (15), that $b_\mathrm{p}(\cdot )$ is bounded and that $\mu _\mathrm{r}\mu _\mathrm{s}<k_\mathrm{r}k_\mathrm{s}(1-e^{-\mu _\mathrm{s}\tau _\mathrm{s}})$. Then the knotweed infestation cannot be eradicated completely by A. itadori.

#### Proof

Suppose eradication occurs, then $S(t)\rightarrow 0$ as $t\rightarrow \infty$. Immediately, we have $R(t)\rightarrow 0$ from (13) with (15), $L(t)\rightarrow 0$ from (5) using that $b_\mathrm{p}(\cdot )$ is bounded, and $A(t)\rightarrow 0$ from (7). However, by Theorem 3.1, in the absence of *A. itadori*, the knotweed biomass grows unboundedly under the condition $\mu _\mathrm{r}\mu _\mathrm{s}<k_\mathrm{r}k_\mathrm{s}(1-e^{-\mu _\mathrm{s}\tau _\mathrm{s}})$, giving a contradiction. $\square$

### Unbounded Knotweed Growth in Presence of *A. itadori*

In this subsection we again consider the case when the stem and rhizome production rates are linear and given by (15), but we now introduce biocontrol using the psyllid *A. itadori* whose birth rate function $b_\mathrm{p}(\cdot )$ per stem need not be linear and in fact is kept as a general function satisfying certain properties. We will demonstrate that it is possible for a solution of (6), (7), (12) and (13) to have its four variables *L*(*t*), *A*(*t*), *S*(*t*) and *R*(*t*) growing exponentially with the same growth rate that is lower than in the case, considered in Sect. 3.1, when *A. itadori* is absent. The linear analysis we present here is of a slightly unusual character and is purely formal, but needs careful explanation. It is not linearised analysis about an equilibrium. Rather, it is a linear analysis in which we assume, *a priori*, that a solution exists in which all components have the same exponential growth rate. We exploit the fact that, for such a solution, as $t\rightarrow \infty$ the variables *L* / *S* and *A* / *S* approach constants whose values are not immediately known but are determined later in terms of the growth rate $\lambda$. This approximation linearises the four equations even though the function $b_\mathrm{p}(\cdot )$ is kept general. The result is a characteristic equation for $\lambda$ having an unusual structure. The existence of a positive root under certain conditions is shown thereby confirming, *a-posteriori*, the existence of a solution with the supposed exponential growth.

It makes sense to assume the parameter values are such that the *A. itadori* population will grow in the situation when there are few adult *A. itadori* (i.e. *A*(*t*) is low) but unlimited stems ($S(t)\rightarrow \infty$). In this situation, Eq. (7) approximates to

$$\begin{aligned} A'(t)=b_\mathrm{p}'(0)e^{-\mu _\mathrm{l}^{\infty }\tau _\mathrm{p}}A(t-\tau _\mathrm{p})-\mu _{\mathrm{a}} A(t) \end{aligned}$$

The solution *A*(*t*) will grow if the coefficient of the delay term exceeds that of the undelayed term (see Kuang 1993), thus we assume that

19 $$\begin{aligned} b_\mathrm{p}'(0)e^{-\mu _\mathrm{l}^{\infty }\tau _\mathrm{p}}>\mu _{\mathrm{a}}. \end{aligned}$$

We suppose the existence of a solution of (6), (7), (12) and (13) such that

$$\begin{aligned} (L(t),A(t),S(t),R(t)) \sim (c_1,c_2,c_3,c_4)\exp (\lambda t) \quad \text{ as } t\rightarrow \infty \end{aligned}$$

with $\lambda >0$ and $c_i>0,i=1,\ldots ,4$. Using expression (11) for *S*(*t*), and the linear stem and rhizome production rates (15), we can write

20 $$\begin{aligned} R'(t)=-\mu _\mathrm{r} R(t)+k_\mathrm{r}k_\mathrm{s} \int _{t-\tau _\mathrm{s}}^t R(\xi )e^{-\mu _\mathrm{s}(t-\xi )} \exp \left( -\int _{\xi }^t\frac{e\sigma L(\eta )}{1+he\sigma S(\eta )}\,\hbox {d}\eta \right) \hbox {d}\xi . \nonumber \\ \end{aligned}$$

For large times, $e\sigma L(t)/(1+he\sigma S(t))\sim c_1/(c_3 h)$ and so (20) becomes, approximately,

21 $$\begin{aligned} R'(t)=-\mu _\mathrm{r} R(t)+k_\mathrm{r}k_\mathrm{s} \int _{0}^{\tau _\mathrm{s}} R(t-\eta )e^{-\left( \mu _\mathrm{s}+\frac{c_1}{c_3 h}\right) \eta }\,\hbox {d}\eta . \end{aligned}$$

With $R(t)=c_4e^{\lambda t}$, this becomes

22 $$\begin{aligned} \lambda +\mu _\mathrm{r}=k_\mathrm{r}k_\mathrm{s}\left( \frac{1-e^{-\left( \lambda +\mu _\mathrm{s}+\frac{c_1}{c_3 h}\right) \tau _\mathrm{s}}}{\lambda +\mu _\mathrm{s}+\frac{c_1}{c_3 h}}\right) . \end{aligned}$$

Also, as the variables get very large, $A(t)/S(t)\sim c_2/c_3$ and $\mu _\mathrm{l}(S(t))\sim \mu _\mathrm{l}^{\infty }$ and so the integral expression (5) for *L*(*t*) becomes, approximately,

23 $$\begin{aligned} L(t)=b_\mathrm{p}\left( \frac{c_2}{c_3}\right) \int _0^{\tau _\mathrm{p}}S(t-\eta )e^{-\mu _\mathrm{l}^{\infty }\eta }\,\hbox {d}\eta \end{aligned}$$

which, with $L(t)=c_1e^{\lambda t}$ and $S(t)=c_3e^{\lambda t}$, becomes

24 $$\begin{aligned} \frac{c_1}{c_3}=b_\mathrm{p}\left( \frac{c_2}{c_3}\right) \left( \frac{1-e^{-(\lambda +\mu _\mathrm{l}^{\infty })\tau _\mathrm{p}}}{\lambda +\mu _\mathrm{l}^{\infty }}\right) . \end{aligned}$$

Finally, we need information about $c_2/c_3$. This will be obtained from the equation for *A*(*t*), Eq. (7). As $t\rightarrow \infty ,A(t)/S(t)\sim c_2/c_3$ and so (7) becomes, approximately,

25 $$\begin{aligned} A'(t)=e^{-\mu _\mathrm{l}^{\infty }\tau _\mathrm{p}}\,b_\mathrm{p}\left( \frac{c_2}{c_3}\right) S(t-\tau _\mathrm{p})-\mu _{\mathrm{a}} A(t). \end{aligned}$$

With $A(t)=c_2e^{\lambda t}$ and $S(t)=c_3e^{\lambda t}$, this becomes

$$\begin{aligned} (\lambda +\mu _{\mathrm{a}})\frac{c_2}{c_3}=e^{-(\lambda +\mu _\mathrm{l}^{\infty })\tau _\mathrm{p}}\,b_\mathrm{p}\left( \frac{c_2}{c_3}\right) \end{aligned}$$

which implicitly determines $c_2/c_3$. So, we let $\alpha (\lambda )>0$ be defined as the solution (existence of which will be shown below) of

26 $$\begin{aligned} (\lambda +\mu _{\mathrm{a}})\alpha (\lambda )=e^{-(\lambda +\mu _\mathrm{l}^{\infty })\tau _\mathrm{p}}\,b_\mathrm{p}(\alpha (\lambda )). \end{aligned}$$

Then $c_2/c_3=\alpha (\lambda )$ and $c_1/c_3$ is determined in terms of $\alpha (\lambda )$ and $\lambda$ using (24). Substituting the result into (22), we obtain the following characteristic equation to be solved for $\lambda$:

27 $$\begin{aligned} \lambda +\mu _\mathrm{r} = k_\mathrm{r}k_\mathrm{s}\left( \frac{1-\exp \left( -\left\{ \lambda +\mu _\mathrm{s}+ b_\mathrm{p}(\alpha (\lambda ))\left( \frac{1-e^{-(\lambda +\mu _\mathrm{l}^{\infty })\tau _\mathrm{p}}}{h(\lambda +\mu _\mathrm{l}^{\infty })}\right) \right\} \tau _\mathrm{s}\right) }{\lambda +\mu _\mathrm{s}+b_\mathrm{p}(\alpha (\lambda ))\left( \frac{1-e^{-(\lambda +\mu _\mathrm{l}^{\infty })\tau _\mathrm{p}}}{h(\lambda +\mu _\mathrm{l}^{\infty })}\right) }\right) \end{aligned}$$

with $\alpha (\lambda )$ defined by (26). As regards existence of $\alpha (\lambda )>0$, we are only assured of this for small positive $\lambda$. Though zero is not an admissible value for $\lambda$ in this subsection, existence of $\alpha (0)$ nevertheless follows from (19) and the other properties of $b_\mathrm{p}(\cdot )$. Continuity arguments then yield existence of $\alpha (\lambda )>0$ for small positive $\lambda$. Moreover, $\alpha (\lambda )$ is decreasing in $\lambda$ for sufficiently small $\lambda$.

As a check on (27), we can let $h\rightarrow \infty$. Recall that *h* is the larva’s digestion time per unit biomass of sap consumed. Increasing this parameter should make the *A. itadori* larvae less effective as a biocontrol agent. Indeed, in the limit as $h\rightarrow \infty$, (27) formally tends to the earlier characteristic equation (18) for the case when *A. itadori* is absent.

Recall that the foregoing analysis presumes that $\lambda$ is a small positive number. Continuity arguments yield the existence of a small positive root of (27) if

28 $$\begin{aligned} \mu _\mathrm{r} <_\mathrm{sl} k_\mathrm{r}k_\mathrm{s}\left( \frac{1-\exp \left( -\left\{ \mu _\mathrm{s}+ b_\mathrm{p}(\alpha (0))\left( \frac{1-e^{-\mu _\mathrm{l}^{\infty }\tau _\mathrm{p}}}{h\mu _\mathrm{l}^{\infty }}\right) \right\} \tau _\mathrm{s}\right) }{\mu _\mathrm{s}+b_\mathrm{p}(\alpha (0))\left( \frac{1-e^{-\mu _\mathrm{l}^{\infty }\tau _\mathrm{p}}}{h\mu _\mathrm{l}^{\infty }}\right) }\right) \end{aligned}$$

where $<_\mathrm{sl}$ means slightly less than, because if (28) holds then the graphs of the left- and right-hand sides of (27), as functions of $\lambda$, must cross each other at some small positive value of $\lambda$. Thus we have formally established the existence of a slowly growing exponential solution of (6), (7), (12) and (13) under condition (28). Since the analysis presumes that $\lambda >0$, it makes no sense to consider the existence of negative real roots of the characteristic equation (27), and no conclusions can be drawn from the existence thereof. However, in view of the impossibility of solutions tending to zero (Theorem 3.2), the *nonexistence* of positive roots of (27) could suggest that solutions remain bounded and this would be an important point in relation to the issue of effective biocontrol using *A. itadori*. We therefore *conjecture* that if $\mu _\mathrm{r}$ exceeds the right-hand side of (28) then solutions remain bounded. In terms of the parameter *h*, which measures the insect’s digestion time per unit biomass consumed, intuitively we expect that effective biocontrol with bounded solutions will only happen for *h* below some threshold, with solutions growing exponentially otherwise. The function $x\rightarrow (1-e^{-x})/x$ is decreasing in *x*, and therefore the right-hand side of (28) increases with *h* (note that $\alpha (0)$ does not involve *h*). This leads us to make the following conjecture.

#### Conjecture 3.3

If $h<h_\mathrm{crit}$, where $h_\mathrm{crit}$ satisfies

29 $$\begin{aligned} \mu _\mathrm{r} = k_\mathrm{r}k_\mathrm{s}\left( \frac{1-\exp \left( -\left\{ \mu _\mathrm{s}+ b_\mathrm{p}(\alpha (0))\left( \frac{1-e^{-\mu _\mathrm{l}^{\infty }\tau _\mathrm{p}}}{\mu _\mathrm{l}^{\infty }h_\mathrm{crit}}\right) \right\} \tau _\mathrm{s}\right) }{\mu _\mathrm{s}+b_\mathrm{p}(\alpha (0))\left( \frac{1-e^{-\mu _\mathrm{l}^{\infty }\tau _\mathrm{p}}}{\mu _\mathrm{l}^{\infty }h_\mathrm{crit}}\right) }\right) \end{aligned}$$

then solutions of (6), (7), (12) and (13) remain bounded.

The true threshold value of *h* distinguishing between boundedness and exponential growth turns out to be higher than $h_\mathrm{crit}$, as we demonstrate later through numerical simulations. Again it is stressed that the foregoing analysis is purely formal.

### Equilibrium Population for Small *h*

In this section, we assume parameters are such that the knotweed would grow exponentially in the absence of *A. itadori*. We show that, in the presence of *A. itadori*, if *h* is sufficiently small then solutions may evolve to a stable equilibrium so that the predator controls, but does not eradicate, the knotweed. No equilibrium exists if *h* is large. Therefore, *A. itadori* can only stabilize the knotweed population if it is able to digest the knotweed biomass sufficiently quickly. Otherwise, *A. itadori* can only slow down the rate of growth of the knotweed stand which still grows without bound. First note that, if (15) holds, the components of any equilibrium $(L^*,A^*,S^*,R^*)$ satisfy

30 $$\begin{aligned}&\left( \mu _\mathrm{s}+\frac{e\sigma L^*}{1+he\sigma S^*}\right) S^* = k_\mathrm{s} R^*\left( 1-e^{-\mu _\mathrm{s}\tau _\mathrm{s}}\exp \left( -\frac{\tau _\mathrm{s} e\sigma L^*}{1+he\sigma S^*}\right) \right) , \end{aligned}$$

31 $$\begin{aligned}&k_\mathrm{r} S^* = \mu _\mathrm{r} R^*, \end{aligned}$$

32 $$\begin{aligned}&\mu _\mathrm{l}(S^*)L^* = S^*b_\mathrm{p}\left( \frac{A^*}{S^*}\right) \left( 1-e^{-\tau _\mathrm{p}\mu _\mathrm{l}(S^*)}\right) , \end{aligned}$$

33 $$\begin{aligned}&S^*b_\mathrm{p}\left( \frac{A^*}{S^*}\right) e^{-\tau _\mathrm{p}\mu _\mathrm{l}(S^*)} = \mu _{\mathrm{a}} A^*. \end{aligned}$$

Let the function $\varphi (S)$ be defined as the positive root of the equation

34 $$\begin{aligned} b_\mathrm{p}(\varphi (S))e^{-\tau _\mathrm{p}\mu _\mathrm{l}(S)}=\mu _{\mathrm{a}}\varphi (S). \end{aligned}$$

A suitable, and biologically realistic, assumption which guarantees that $\varphi (S)$ is well defined for each $S>0$ is (35) below:

35 $$\begin{aligned} {\left\{ \begin{array}{ll} b_\mathrm{p}(0)=0\; \hbox {and, for each fixed}\; S>0,b_\mathrm{p}(x)e^{-\tau _\mathrm{p}\mu _\mathrm{l}(S)}>\mu _{\mathrm{a}} x\; \hbox {for positive}\; x\\ \hbox {up to some threshold, while}\; b_\mathrm{p}(x)e^{-\tau _\mathrm{p}\mu _\mathrm{l}(S)}<\mu _{\mathrm{a}} x\; \hbox {when}\; x\; \hbox {exceeds this}\\ \hbox {threshold.} \end{array}\right. } \end{aligned}$$

Assumption (35) basically states that the maturation rate per stem of *A. itadori* exceeds the death rate per stem if the ratio of adult insects to stems is low, but the opposite is true for high values of this ratio. The threshold in (35) is what we call $\varphi (S)$. It is useful to keep in mind the realistic choice $b_\mathrm{p}(x)=Px e^{-qx}$ mentioned earlier, which satisfies (35) for large enough *P*.

Recall that the function $\mu _\mathrm{l}(S)$ decreases from $\mu _\mathrm{l}^0$ at $S=0$ to $\mu _\mathrm{l}^{\infty }$ at $S=\infty$. If (35) holds, calculus arguments show that $\varphi (S)$ is well defined for any $S>0$, and that $\varphi (S)$ increases with *S* and is confined between two finite positive values. Note also that $\varphi (S^*)=A^*/S^*$. We prove the following result on the existence and stability of a positive equilibrium satisfying (30)–(33) for sufficiently small *h*.

#### Theorem 3.4

Suppose that (15) and (35) hold, and that $\mu _\mathrm{r}\mu _\mathrm{s}<k_\mathrm{r}k_\mathrm{s}(1-e^{-\mu _\mathrm{s}\tau _\mathrm{s}})$ (i.e. knotweed grows in the absence of A. itadori). Then, if *h* is sufficiently small, Eqs. (6), (7), (12) and (13) admit an equilibrium $(L^*,A^*,S^*,R^*)$ in which each component is strictly positive. Moreover, if $\mu _{\mathrm{a}}>b_\mathrm{p}'(\varphi (S^*))e^{-\tau _\mathrm{p}\mu _\mathrm{l}(S^*)},b_\mathrm{p}'(\varphi (S^*))>0$ and $b_\mathrm{p}(\varphi (S^*))-\varphi (S^*)b_\mathrm{p}'(\varphi (S^*))>0$, where $\varphi (S)$ is defined by (34), then any real roots of the characteristic equation of the linearisation about this equilibrium must be negative. If *h* is too large then no such equilibrium exists.

Before we present the proof, we point out that the condition $\mu _{\mathrm{a}}>b_\mathrm{p}'(\varphi (S^*))e^{-\tau _\mathrm{p}\mu _\mathrm{l}(S^*)}$ almost follows from (35) but is added to exclude the unlikely possibility of tangency. The condition $b_\mathrm{p}'(\varphi (S^*))>0$ states that the ratio $A^*/S^*$ must be on the increasing side of any non-monotone $b_\mathrm{p}(\cdot )$, while $b_\mathrm{p}(\varphi (S^*))-\varphi (S^*)b_\mathrm{p}'(\varphi (S^*))>0$ will hold if $b_\mathrm{p}(\cdot )$ is concave ($b_\mathrm{p}''<0$) in the region of interest, which is realistic for the types of choices we have in mind.

#### Proof

Combining (30) and (31) and requiring $S^*>0$ gives

36 $$\begin{aligned} \mu _\mathrm{s}+\frac{e\sigma L^*}{1+he\sigma S^*} = \frac{k_\mathrm{r}k_\mathrm{s}}{\mu _\mathrm{r}}\left( 1-\exp \left\{ -\tau _\mathrm{s}\left( \mu _\mathrm{s}+\frac{e\sigma L^*}{1+he\sigma S^*}\right) \right\} \right) . \end{aligned}$$

Let $c=c^*$ be the root of the equation

37 $$\begin{aligned} \mu _\mathrm{s}+c=\frac{k_\mathrm{r}k_\mathrm{s}}{\mu _\mathrm{r}}[1-\exp (-(\mu _\mathrm{s}+c)\tau _\mathrm{s})]. \end{aligned}$$

Since $\mu _\mathrm{r}\mu _\mathrm{s}<k_\mathrm{r}k_\mathrm{s}(1-e^{-\mu _\mathrm{s}\tau _\mathrm{s}})$ it is easily checked that $c^*>0$ exists and is unique. Note that $e\sigma L^*/(1+he\sigma S^*)=c^*$ and $A^*/S^*=\varphi (S^*)$. Therefore, from (32) we obtain a single equation for the equilibrium component $S^*$:

38 $$\begin{aligned} \mu _\mathrm{l}(S^*)(1+he\sigma S^*)\frac{c^*}{e\sigma }=S^*b_\mathrm{p}(\varphi (S^*))(1-e^{-\tau _\mathrm{p}\mu _\mathrm{l}(S^*)}). \end{aligned}$$

The other equilibrium components are then determined in terms of $S^*$ from

39 $$\begin{aligned} A^*=S^*\varphi (S^*), L^*=(1+he\sigma S^*)\frac{c^*}{e\sigma },R^*=\frac{k_\mathrm{r} S^*}{\mu _\mathrm{r}}. \end{aligned}$$

The existence or otherwise of a root $S^*>0$ of (38) can be considered from graphical arguments. Imagine the left- and right-hand sides are plotted against $S^*$. If $h=0$ the left-hand side of (38), as a function of $S^*>0$, decreases monotonically to a limit $\mu _\mathrm{l}^{\infty }c^*/(e\sigma )$, while the right-hand side starts at 0 and grows without bound, becoming linear in $S^*$ for $S^*$ large. Therefore a root $S^*>0$ exists for $h=0$. If $h>0$ is small, then the left-hand side initially decreases with $S^*$ but eventually starts to increase becoming approximately linear with a low growth rate of $\mu _\mathrm{l}^{\infty }hc^*$ so that again (38) can be solved for a positive $S^*$. However, if *h* is sufficiently large then the left-hand side increases for all $S^*>0$, again becoming roughly linear but with a steeper growth rate than the right-hand side. This prevents the graphs from having an intersection and so there is no equilibrium.

To investigate the linear stability of the equilibrium that exists for small *h*, we set $L=L^*+\tilde{L},A=A^*+\tilde{A},S=S^*+\tilde{S}$ and $R=R^*+\tilde{R}$. Collecting the linear parts of the resulting equations in terms of the new variables, and making use of the equilibrium equations (30)–(33), we obtain

40 $$\begin{aligned} \tilde{L}(t)= & {} \displaystyle {\int _0^{\tau _\mathrm{p}}}e^{-\mu _\mathrm{l}(S^*)\theta }\bigg \{\left[ b_\mathrm{p}(\varphi (S^*)) -\varphi (S^*)b_\mathrm{p}'(\varphi (S^*))\right] \tilde{S}(t-\theta ) \nonumber \\ \quad&+\, b_\mathrm{p}'(\varphi (S^*))\tilde{A}(t-\theta )-\mu _\mathrm{l}'(S^*)S^*b_\mathrm{p}(\varphi (S^*)) \displaystyle {\int _{t-\theta }^t} \tilde{S}(\eta )\,\hbox {d}\eta \bigg \}\,d\theta , \end{aligned}$$

41 $$\begin{aligned} \tilde{A}'(t)= & {} -\mu _{\mathrm{a}} \tilde{A}(t)+e^{-\tau _\mathrm{p}\mu _\mathrm{l}(S^*)}\left[ b_\mathrm{p}(\varphi (S^*)) -\varphi (S^*)b_\mathrm{p}'(\varphi (S^*))\right] \tilde{S}(t-\tau _\mathrm{p}) \nonumber \\&+\, e^{-\tau _\mathrm{p}\mu _\mathrm{l}(S^*)}b_\mathrm{p}'(\varphi (S^*))\tilde{A}(t-\tau _\mathrm{p})\nonumber \\&-\,\mu _\mathrm{l}'(S^*)S^*b_\mathrm{p}(\varphi (S^*))e^{-\tau _\mathrm{p}\mu _\mathrm{l}(S^*)} \displaystyle {\int _0^{\tau _\mathrm{p}}}\tilde{S}(t-\theta )\,d\theta , \end{aligned}$$

42 $$\begin{aligned} \tilde{S}(t)= & {} k_\mathrm{s}\int _0^{\tau _\mathrm{s}}\exp \left( -\left\{ \mu _\mathrm{s}+\frac{e\sigma L^*}{1+he\sigma S^*}\right\} \theta \right) \bigg [ R(t-\theta ) \nonumber \\&-\, \frac{R^*}{1+he\sigma S^*}\int _{t-\theta }^t\left\{ e\sigma \tilde{L}(\eta )- \frac{he^2\sigma ^2 L^*}{1+he\sigma S^*}\,\tilde{S}(\eta )\right\} \hbox {d}\eta \bigg ]\,d\theta , \end{aligned}$$

and

43 $$\begin{aligned} \tilde{R}'(t)=k_\mathrm{r}\tilde{S}(t)-\mu _\mathrm{r}\tilde{R}(t). \end{aligned}$$

Solutions are sought of the form $(\tilde{L},\tilde{A},\tilde{S},\tilde{R})=(d_1,d_2,d_3,d_4)e^{\lambda t}$. This leads, after some immensely complicated algebra, to a characteristic equation to be solved for $\lambda$. Letting

44 $$\begin{aligned} g(x)=\frac{1-e^{-x}}{x} \end{aligned}$$

and casting the characteristic equation in a form where $L^*,A^*$ and $R^*$ have been eliminated, the result is

45 $$\begin{aligned} F_3(\lambda )=-\frac{F_1(\lambda )F_4(\lambda )F_5(\lambda )}{F_2(\lambda )F_5(\lambda )+F_6(\lambda )} \end{aligned}$$

where

46 $$\begin{aligned} F_1(\lambda )= & {} b_\mathrm{p}'(\varphi (S^*))\tau _\mathrm{p} g(\tau _\mathrm{p}(\lambda +\mu _\mathrm{l}(S^*))), \end{aligned}$$

47 $$\begin{aligned} F_2(\lambda )= & {} (b_\mathrm{p}(\varphi (S^*))-\varphi (S^*)b_\mathrm{p}'(\varphi (S^*)))\tau _\mathrm{p} g(\tau _\mathrm{p}(\lambda +\mu _\mathrm{l}(S^*))) \nonumber \\&-\mu _\mathrm{l}'(S^*)S^*b_\mathrm{p}(\varphi (S^*))\tau _\mathrm{p}\left( \frac{g(\tau _\mathrm{p}\mu _\mathrm{l}(S^*))-g(\tau _\mathrm{p}(\lambda +\mu _\mathrm{l}(S^*)))}{\lambda }\right) , \end{aligned}$$

48 $$\begin{aligned} F_3(\lambda )= & {} \lambda +\mu _{\mathrm{a}}-b_\mathrm{p}'(\varphi (S^*))e^{-\tau _\mathrm{p}(\lambda +\mu _\mathrm{l}(S^*))}, \end{aligned}$$

49 $$\begin{aligned} F_4(\lambda )= & {} e^{-\tau _\mathrm{p}(\lambda +\mu _\mathrm{l}(S^*))}(b_\mathrm{p}(\varphi (S^*))-\varphi (S^*)b_\mathrm{p}'(\varphi (S^*))) \nonumber \\&-\mu _\mathrm{l}'(S^*)S^*b_\mathrm{p}(\varphi (S^*))\tau _\mathrm{p} e^{-\tau _\mathrm{p}\mu _\mathrm{l}(S^*)}g(\tau _\mathrm{p}\lambda ), \end{aligned}$$

50 $$\begin{aligned} F_5(\lambda )= & {} \frac{k_\mathrm{r}k_\mathrm{s} S^* e\sigma \tau _\mathrm{s}}{\mu _\mathrm{r}(1+he\sigma S^*)}\left( \frac{g(\tau _\mathrm{s}(\mu _\mathrm{s}+c^*))-g(\tau _\mathrm{s}(\lambda +\mu _\mathrm{s}+c^*))}{\lambda }\right) , \end{aligned}$$

51 $$\begin{aligned} F_6(\lambda )= & {} 1-\frac{k_\mathrm{r}k_\mathrm{s}\tau _\mathrm{s}}{\lambda +\mu _\mathrm{r}}g(\tau _\mathrm{s}(\lambda +\mu _\mathrm{s}+c^*)) \nonumber \\&-\frac{k_\mathrm{r}k_\mathrm{s} S^* he\sigma c^*\tau _\mathrm{s}}{\mu _\mathrm{r}(1+he\sigma S^*)}\left( \frac{ g(\tau _\mathrm{s}(\mu _\mathrm{s}+c^*))-g(\tau _\mathrm{s}(\lambda +\mu _\mathrm{s}+c^*))}{\lambda }\right) , \end{aligned}$$

where we have used (39) and we remind the reader that $c^*$ is the unique positive root of (37). Let us consider the behaviour of the above expressions for $\lambda \ge 0$. Now $g(x)=(1-e^{-x})/x$ is a positive decreasing function which satisfies $g''(x)>0$. Also $\mu _\mathrm{l}(\cdot )$ is decreasing so that $\mu _\mathrm{l}'(S^*)<0$. Moreover $b_\mathrm{p}'(\varphi (S^*))>0$ and $b_\mathrm{p}(\varphi (S^*))-\varphi (S^*)b_\mathrm{p}'(\varphi (S^*))>0$. Using these facts, calculus arguments yield that the functions $F_1(\lambda ),\ldots ,F_6(\lambda )$ have the following properties for $\lambda \ge 0$: $F_1(\lambda ),F_2(\lambda ),F_4(\lambda )$ and $F_5(\lambda )$ are positive and decreasing to zero as $\lambda \rightarrow \infty ,F_3(\lambda )$ is increasing, $F_6(\lambda )\rightarrow 1$ as $\lambda \rightarrow \infty$, and is increasing if *h* is sufficiently small.

We claim that the denominator $F_2(\lambda )F_5(\lambda )+F_6(\lambda )$ of (45) is positive for all $\lambda \ge 0$ if *h* is sufficiently small. The first two terms in the right-hand side of (51) cancel out when $\lambda =0$, due to the fact that $c^*$ satisfies (37). Therefore, for small $h,F_6(0)$ is a small negative number of *O*(*h*), while $F_2(0)$ and $F_5(0)$ are positive and *O*(1) in *h*. So $F_2(0)F_5(0)+F_6(0)>0$ for sufficiently small *h*. Also $F_2(\lambda )F_5(\lambda )+F_6(\lambda )\rightarrow 1$ as $\lambda \rightarrow \infty$, so the question is whether $F_2(\lambda )F_5(\lambda )+F_6(\lambda )$ can go negative for some intermediate $\lambda >0$. Since $F_6(\lambda )$ is increasing, this evidently cannot happen after $F_6(\lambda )$ has become positive so we need only concern ourselves with a small compact interval near $\lambda =0$ where $F_6(\lambda )<0$. In every point of this interval, $F_6(\lambda )$ is small and of *O*(*h*), while $F_2(\lambda )$ and $F_5(\lambda )$ are positive and *O*(1) in *h*. Therefore, for sufficiently small $h,F_2(\lambda )F_5(\lambda )+F_6(\lambda )>0$ throughout the small interval near $\lambda =0$ where $F_6(\lambda )<0$. It should be recalled that $S^*$ depends on *h*, however $S^*$ approaches a strictly positive value as $h\rightarrow 0$.

We now have that, if *h* is sufficiently small, the right-hand side of (45) is negative for all $\lambda \ge 0$. The left-hand side is increasing and is therefore positive for all $\lambda \ge 0$, since $F_3(0)=\mu _{\mathrm{a}}-b_\mathrm{p}'(\varphi (S^*))e^{-\tau _\mathrm{p}\mu _\mathrm{l}(S^*)}>0$ by hypothesis. Therefore, any real roots of (45) must be negative and the proof is complete. $\square$

## Numerical Simulations

In this section, we present the results of numerical simulations which confirm the analytical results of Sect. 3, and yield evidence for the truth of Conjecture 3.3.

We assume that the decreasing function $\mu _\mathrm{l}(S)$ is given by $\mu _\mathrm{l}(S)=k_\mathrm{l}e^{-S}+m$, that $b_\mathrm{p}(x)=Px e^{-qx}$, and we use the following parameter values:

52 $$\begin{aligned} \begin{array}{ll} &{}m=0.0205, P=16.9867, q=1, \mu _{\mathrm{a}}=0.0806, e=1, \sigma =1, \\ &{}k_\mathrm{s}=0.1, k_\mathrm{r}=0.1, \mu _\mathrm{r}=0.01, \mu _\mathrm{s}=0.01, \tau _\mathrm{p}=32.2, \tau _\mathrm{s}=150, \end{array} \end{aligned}$$

and the following initial estimate $A^0$ of the number of adult *A. itadori* and quantity $R^0$ of rhizome biomass:

53 $$\begin{aligned} A^0=50, \;\; R^0=500. \end{aligned}$$

For a complete list of the symbols and parameter values used, see Table 1. With the parameter values (52), we have

54 $$\begin{aligned} \alpha _0=4.6906 \end{aligned}$$

and

55 $$\begin{aligned} h_{\text {crit}}\approx 17.4172, \end{aligned}$$

where the unit of $h_\mathrm{crit}$ is the number of days per unit of stem biomass per larva.

**Fig. 1:**
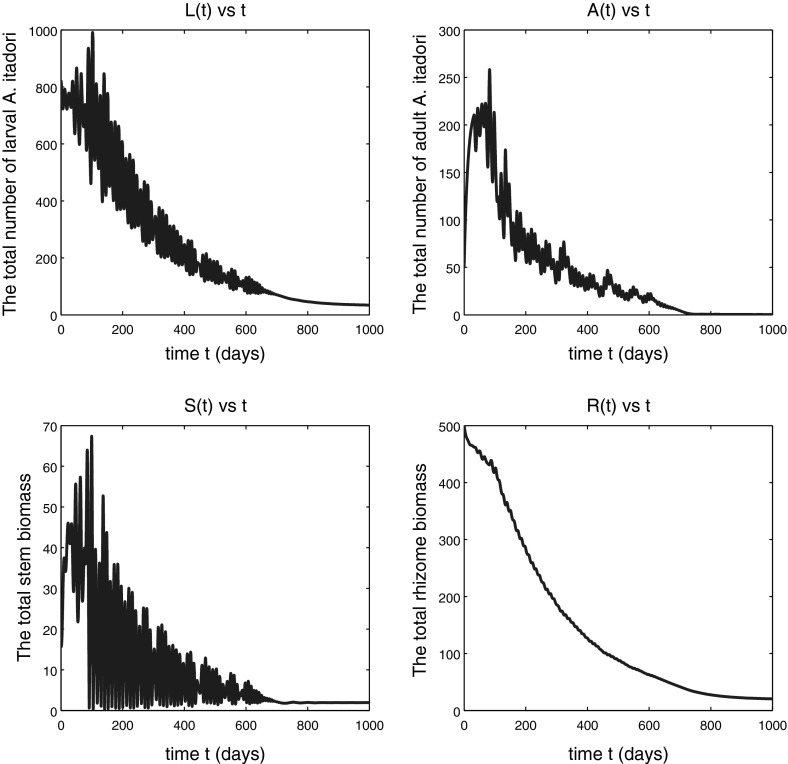
Solutions of model (6), (7), (12) and (13) subject to the initial conditions (14). The *top panel* shows the numbers of larval (*left*) and adult (*right*) *A. itadori* against time. The *bottom panel* shows total *F. japonica* stem (*left*) and rhizome (*right*) biomass against time. Parameter values used are given in (52) and $h=h_{\text {crit}}-1$. The variable *A*(*t*) tends to a small positive limit, not to zero, as $t\rightarrow \infty$

**Fig. 2:**
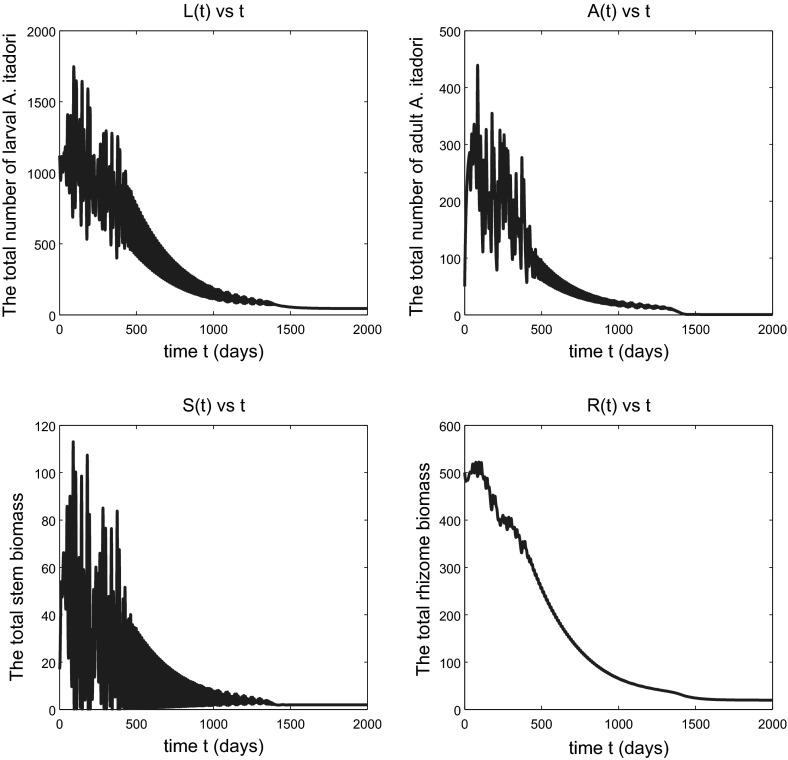
Solutions of model (6), (7), (12) and (13) subject to the initial conditions (14). Parameter values used are given in (52) and $h=h_{\text {crit}}+5$. The variable *A*(*t*) tends to a small positive limit, not to zero, as $t\rightarrow \infty$

If $h<h_{\text {crit}}$, we expect that the solutions remain bounded (Conjecture 3.3). Figure 1 shows that the total numbers of larval and adult *A. itadori* approach equilibrium values after a long oscillatory transient of around 3 years, with higher numbers of larval than adult *A. itadori* at equilibrium. The number of adults *A*(*t*) approaches a very small positive limit, not zero, and Fig. 1 therefore suggests that effective long-term control of *F. japonica* by *A. itadori* is possible with surprisingly few adult *A. itadori*. The oscillatory nature of the transient is not surprising since this is a predator–prey interaction between *A. itadori* larvae (the predator) and the *F. japonica* stems (the prey). The simulation shown in Fig. 2 is for a situation with $h>h_{\text {crit}}$ but again indicates slow convergence to equilibrium, this time on an even longer timescale. Thus, $h_{\text {crit}}$ is not a true threshold value for *h*; bounded or convergent behaviour is possible even for some $h>h_{\text {crit}}$ (though not for all *h*). A detailed study of the transient region of Fig. 2 showed that the fluctuations are on a cycle of roughly 12 days, about the same as the mean adult longevity $1/\mu _{\mathrm{a}}$ of adult *A. itadori* with the value for $\mu _{\mathrm{a}}$ in (52). The bottom panel of Fig. 2 shows that both total stem biomass and total rhizome biomass approach equilibrium values after long transients. The variable *S* representing total stem biomass has a much higher amplitude of oscillation than the rhizome during the transient, no doubt because *A. itadori* eats only the stems. Mathematically, a lower amplitude of oscillation for rhizome biomass *R* can be anticipated because the solution *R*(*t*) of (13) at time *t* will involve a weighted average of past values of the stem biomass variable *S*.

**Fig. 3:**
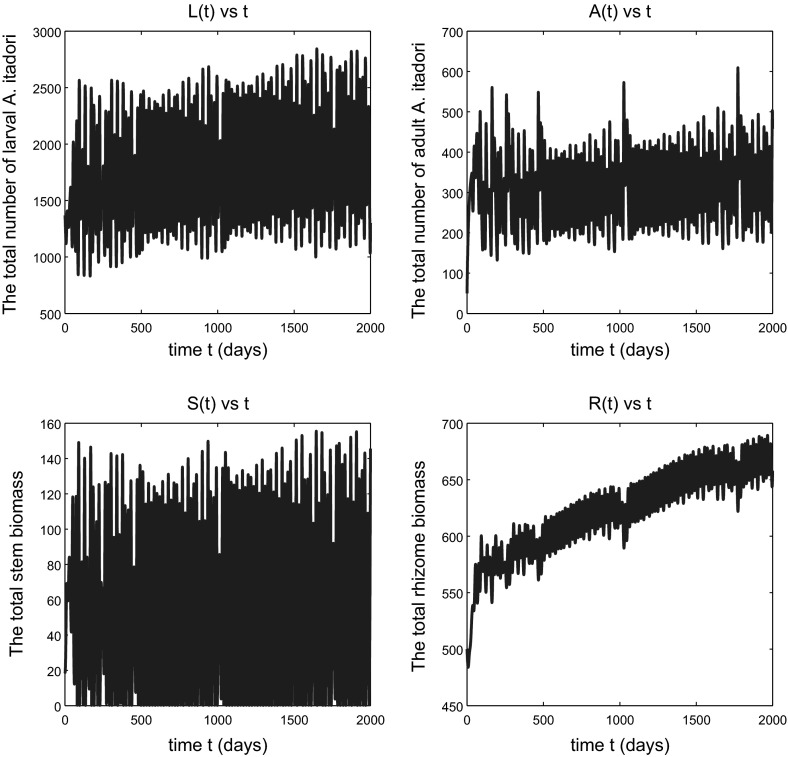
Solutions of model (6), (7), (12) and (13) subject to the initial conditions (14). Parameter values used are given in (52) and $h=h_{\text {crit}}+10$

**Fig. 4:**
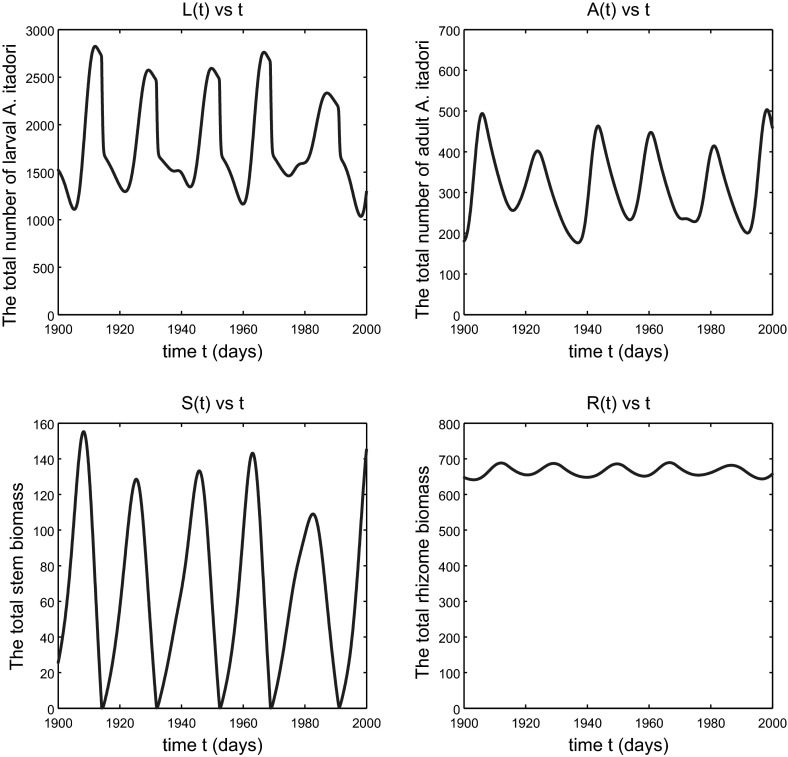
The situation of Fig. 3 showing the detail of the sustained oscillation

**Fig. 5:**
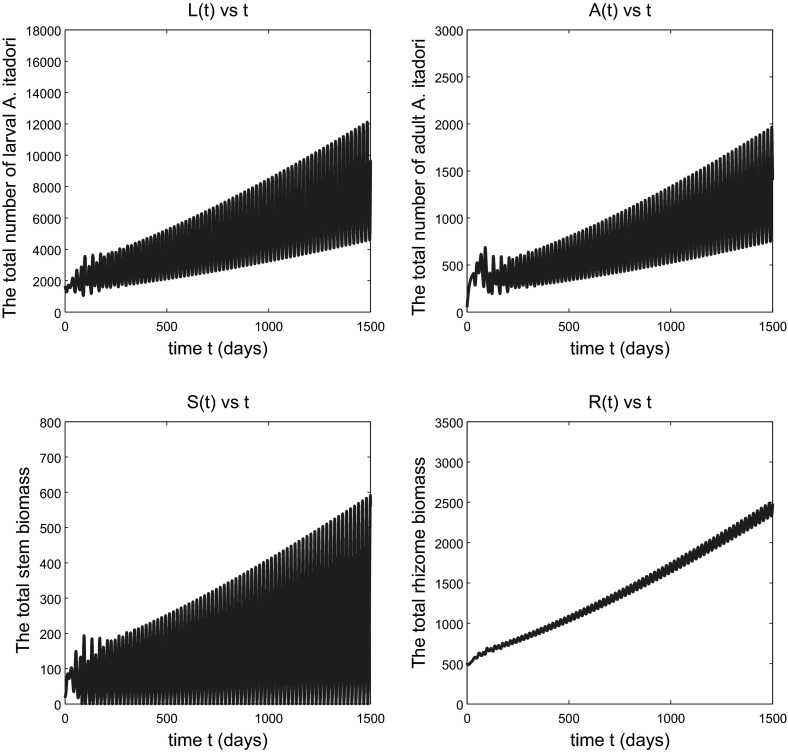
Solutions of model (6), (7), (12) and (13) subject to the initial conditions (14). Parameter values used are given in (52) and $h=h_{\text {crit}}+15$

As we anticipated in the comment after Conjecture 3.3, which has been confirmed by the simulation shown in Fig. 2, when *h* is slightly above $h_{\text {crit}}$ the total *F. japonica* stem and rhizome biomass variables remain bounded; in fact they continue to approach equilibrium values for *h* sufficiently close to $h_{\text {crit}}$. So the weed continues to be effectively controlled by *A. itadori*. Figure 3 shows a situation, for a larger $h>h_{\text {crit}}$, in which the variables still remain bounded but no longer approach equilibrium values. The situation here is one of sustained oscillations, the details of which are revealed in Fig. 4. If *h* is increased sufficiently, we eventually see unbounded oscillatory growth of *F. japonica*, as shown in Fig. 5, in which the peaks of all variables *L*(*t*), *A*(*t*), *S*(*t*) and *R*(*t*) grow in time. The total stem biomass still has a higher amplitude of oscillation than the rhizome. Such findings imply that the true threshold value for *h*, that distinguishes between boundedness and unbounded growth of solution variables, is higher than $h_\mathrm{crit}$. This is not a surprise since most of the parameter values we used for *F. japonica* are based on approximations. All of these results together are very intuitive: if *A. itadori* voraciously consumes the stem biomass of *F. japonica*, then the growth of *F. japonica* is limited. Therefore, using *A. itadori* to control *F. japonica* is possible. However, *F. japonica* grows without bound if *A. itadori* is not sufficiently voracious.

## Discussion

We have developed a model to study the bio-control of *F. japonica* using one of its co-evolved natural enemies, the Japanese sap-sucking psyllid *A. itadori*. Our study focuses on a single isolated knotweed stand by modelling the growth of the stem and the rhizome with the stems under predation by larval *A. itadori*. Our final model consists of four differential equations with time delays subject to initial conditions with constraints. These four equations represent the rates of change of larval and adult *A. itadori*, and stem and rhizome biomass. Our results show that *F. japonica* will grow unboundedly in the absence of *A. itadori* if the natural loss of the stem biomass and the rhizome biomass is low. If such loss is high enough, then the knotweed stand decays exponentially. We have focused on the case when knotweed would grow in the absence of *A. itadori*, showing that in this case *A. itadori* cannot completely eradicate the knotweed but will either slow down its growth or, if it consumes the sap sufficiently voraciously, will cause the stem and rhizome biomass variables to remain bounded and possibly approach equilibrium values. The latter scenarios constitute the most desirable and effective form of biocontrol of knotweed whereby, based on a limited form of linearised analysis and numerical simulations, the knotweed and biocontrol agent coexist at a stable equilibrium or in the form of long-term sustained oscillations about an equilibrium.

It turns out that the dynamics of the model depend mainly on a parameter *h*, which measures how long it takes for an *A. itadori* larva to handle (digest) one unit of *F. japonica* stem biomass. Using model parameters based on the best available information, we provide an estimate of $h_\mathrm{crit}$. If *h* is too large (i.e. *h* is much larger than $h_\mathrm{crit}$), then the model does not have a positive equilibrium and the plant biomass and insect numbers both grow together without bound, though at a lower rate than if *A. itadori* were absent. On the other hand, if *h* is sufficiently small, then the model implies that the knotweed plant biomass and insect numbers remain bounded and may approach equilibrium values. Rigorous mathematical analysis of our model is very challenging. In our study of the linearised stability of the coexistence equilibrium, we were only able to consider the real roots of the characteristic equation, even though the dominant root need not be real. In these circumstances, of course, negativity of the real roots does not conclusively yield stability of an equilibrium. However, the result on the existence of such an equilibrium for sufficiently small *h* is rigorous and this finding by itself is very important, since the knotweed stand grows without bound if no such equilibrium exists. It might be possible to prove boundedness of solution variables for sufficiently small *h* by other techniques, but our model is intractable to all strategies for proving boundedness that we have tried. Such a rigorous boundedness result for small *h* would be very desirable since the sole aim is to control the knotweed.

In this paper, we did not model the spatial spread of a knotweed stand explicitly, though we noted that the stand must spread in space if total stem biomass is increasing without bound with individual stems only growing to a certain height. Knotweed rhizomes are known to spread very vigorously and Smith et al. (2007) modelled the development of the rhizome system of a single stand using a 3D correlated random walk model. Their model predicts that the area of a stand increases quadratically with time and further suggests that it would be reasonable to model the growth of a stand using a reaction diffusion equation. Data from the spread of existing infestations could help to estimate the diffusivity. Development of our model to include the spread of a stand using such ideas is to be an area of future work. Spatial spread by other mechanisms such as downstream drift of root fragments (e.g., Hugh Dawson and Holland 1999) will also be important in future modelling efforts.

## Appendix

In this section we explain how we performed the numerical simulations for the model consisting of Eqs. (6), (7), (12) and (13), subject to the initial conditions (14). Recall that we assume $\mu _\mathrm{l}(S)=k_\mathrm{l}e^{-S}+m$ and $b_\mathrm{p}(x)=Px e^{-qx}$.

Equations (6), (7) and (12) contain distributed delayed terms, and the numerical simulation is more easily conducted with the aid of the auxiliary functions

56 $$\begin{aligned} X(t)\triangleq & {} \exp \left( -\displaystyle {\int _{t-\tau _\mathrm{p}}^t}\mu _\mathrm{l}(S(\eta ))\,\hbox {d}\eta \right) ,\end{aligned}$$

57 $$\begin{aligned} Y(t)\triangleq & {} \exp \left( -\int _{t-\tau _\mathrm{s}}^t\frac{e\sigma L(\eta )\,\hbox {d}\eta }{1+he\sigma S(\eta )} \right) . \end{aligned}$$

The derivatives of *X*(*t*) and *Y*(*t*) are given by

58 $$\begin{aligned} \frac{\hbox {d}X(t)}{\hbox {d}t}= & {} X(t)\bigg (-\mu _\mathrm{l}(S(t))+\mu _\mathrm{l}(S(t-\tau _\mathrm{p}))\bigg ), \end{aligned}$$

59 $$\begin{aligned} \frac{\hbox {d}Y(t)}{\hbox {d}t}= & {} Y(t)\left( -\frac{e\sigma L(t)}{1+he \sigma S(t)}+\frac{e \sigma L(t-\tau _\mathrm{s})}{1+h e\sigma S(t-\tau _\mathrm{s})}\right) . \end{aligned}$$

Therefore, we may simulate the following system without distributed delays:

60 $$\begin{aligned} \begin{array}{rcl} \displaystyle {\frac{\hbox {d}L(t)}{\hbox {d}t}} &{} = &{} -\mu _\mathrm{l}(S(t))L(t)+S(t)b_\mathrm{p}\left( \displaystyle {\frac{A(t)}{S(t)}}\right) -S(t-\tau _\mathrm{p})b_\mathrm{p}\left( \displaystyle {\frac{A(t-\tau _\mathrm{p})}{S(t-\tau _\mathrm{p})}}\right) X(t), \\ \displaystyle {\frac{\hbox {d}A(t)}{\hbox {d}t}} &{}=&{}S(t-\tau _\mathrm{p})b_\mathrm{p}\left( \displaystyle {\frac{A(t-\tau _\mathrm{p})}{S(t-\tau _\mathrm{p})}}\right) X(t)-\mu _{\mathrm{a}}A(t),\\ \displaystyle {\frac{\hbox {d}S(t)}{\hbox {d}t}} &{} = &{} -\left( \mu _\mathrm{s}+\displaystyle {\frac{e\sigma L(t)}{1+he\sigma S(t)}}\right) S(t)+b_\mathrm{s}(R(t)) -e^{-\mu _\mathrm{s}\tau _\mathrm{s}}Y(t)b_\mathrm{s}(R(t-\tau _\mathrm{s})),\\ \displaystyle {\frac{\hbox {d}R(t)}{\hbox {d}t}} &{}=&{}b_\mathrm{r}(S(t))-\mu _\mathrm{r} R(t),\\ \displaystyle {\frac{\hbox {d}X(t)}{\hbox {d}t}}&{}=&{} X(t)\bigg (-\mu _\mathrm{l}(S(t))+\mu _\mathrm{l}(S(t-\tau _\mathrm{p})\bigg ),\\ \displaystyle {\frac{\hbox {d}Y(t)}{\hbox {d}t}}&{}=&{} Y(t)\left( -\displaystyle {\frac{e\sigma L(t)}{1+h e\sigma S(t)}}+\displaystyle {\frac{e\sigma L(t-\tau _\mathrm{s})}{1+he\sigma S(t-\tau _\mathrm{s})}}\right) , \end{array} \end{aligned}$$

subject to the initial conditions:

61 $$\begin{aligned} L(\theta )= & {} L^0(\theta )\ge 0, \;\;\; \theta \in [-\tau _\mathrm{s}, 0], \end{aligned}$$

62 $$\begin{aligned} A(\theta )= & {} A^0(\theta )\ge 0, \;\;\; \theta \in [-\tau _\mathrm{p}, 0], \end{aligned}$$

63 $$\begin{aligned} S(\theta )= & {} S^0(\theta )\ge 0, \;\;\; \theta \in [-\max (\tau _\mathrm{p}, \tau _\mathrm{s}), 0], \end{aligned}$$

64 $$\begin{aligned} R(\theta )= & {} R^0(\theta )\ge 0, \;\;\; \theta \in [-\tau _\mathrm{s}, 0], \end{aligned}$$

65 $$\begin{aligned} X(0)= & {} X^0\ge 0, \end{aligned}$$

66 $$\begin{aligned} Y(0)= & {} Y^0\ge 0, \end{aligned}$$

67 $$\begin{aligned} L^0(0)= & {} \int _{-\tau _\mathrm{p}}^{0} S^0(\xi )b_\mathrm{p}(A^0(\xi )/S^0(\xi )) \exp \left( -\int _{\xi }^0\mu _\mathrm{l}(S^0(\eta ))\,\hbox {d}\eta \right) \hbox {d}\xi , \end{aligned}$$

68 $$\begin{aligned} S^0(0)= & {} \int _{-\tau _\mathrm{s}}^0 b_\mathrm{s}(R^0(\xi ))e^{\mu _\mathrm{s}\xi } \exp \left( -\int _{\xi }^0\frac{e\sigma L^0(\eta )}{1+he\sigma S^0(\eta )}\,\hbox {d}\eta \right) \hbox {d}\xi , \end{aligned}$$

69 $$\begin{aligned} X^0= & {} \exp \left( -\int _{-\tau _\mathrm{p}}^0\mu _\mathrm{l}(S^0(\eta ))\,\hbox {d}\eta \right) , \end{aligned}$$

70 $$\begin{aligned} Y^0= & {} \exp \left( -\int _{-\tau _\mathrm{s}}^0\frac{e\sigma L^0(\eta )}{1+he\sigma S^0(\eta )}\,\hbox {d}\eta \right) . \end{aligned}$$

It is necessary to choose appropriate initial conditions which will be used as the history functions when one solves system (60) using dde23 in MATLAB. We choose the initial functions $A^0(\theta )$ and $R^0(\theta )$ to be constants *A* and *R*, respectively, and then use the integral constraints (67)–(70) to find constant initial data for the other variables, on their respective initial intervals, in terms of *A* and *R*. The constraints (67)–(70) determine that the constant initial functions for the variables *L*, *S*, *X*, and *Y*, which are used as the history functions in the MATLAB routine dde23, are the functions identically equal to the real numbers (again denoted *L*, *S*, *X*, and *Y*) that satisfy

71 $$\begin{aligned}&L-PAe^{-q\frac{A}{S}}\frac{1}{k_\mathrm{l}e^{-S}+m}\bigg (1-e^{-(k_\mathrm{l}e^{-S}+m)\tau _\mathrm{p}}\bigg )=0, \end{aligned}$$

72 $$\begin{aligned}&S-k_\mathrm{s}R \frac{1}{\mu _\mathrm{s}+\frac{e\sigma L}{1+he\sigma S}}\bigg (1-e^{-(\mu _\mathrm{s}+\frac{e\sigma L}{1+he\sigma S})\tau _\mathrm{s}}\bigg ) = 0,\end{aligned}$$

73 $$\begin{aligned}&X- \exp \big \{-(k_\mathrm{l}e^{-S}+m)\tau _\mathrm{p}\big \}=0,\end{aligned}$$

74 $$\begin{aligned}&Y- \exp \left\{ -\frac{e\sigma L}{1+he\sigma S}\tau _\mathrm{s}\right\} =0, \end{aligned}$$

for specified values of *A* and *R*. Our simulations are of system (60), with initial data chosen to satisfy the integral constraints as just described.
